# Supplementary material for: Heat adaptation of phage T7 under an extended genetic code
Source: Virus Evol. 2021 Dec 1;7(2):veab100. doi: 10.1093/ve/veab100 (PMC8923235; doi:10.1093/ve/veab100)
Supplement: veab100_Supp [file veab100_supp.zip › Sequencing_Analysis.zip!/Sequencing_Analysis/A1/evidence/RA_37.html]

BRESEQ :: Evidence


| Read alignment evidence... | | | | | | | | | | | |
| --- | --- | --- | --- | --- | --- | --- | --- | --- | --- | --- | --- |
|  | seq id | position | | ref | new | freq | score (cons/poly) | reads | annotation | genes | product |
| \* | NC\_001604.1 | 29,697 | 0 | C | T | 61.0% | 755.1 / inf | 2651 | intergenic (–/–) | *–/–* | –/– |
| Reads supporting (aligned to +/- strand):  **ref** base C (332/277);  **major** base T (696/541);  **minor** base A (450/341);  **total** (1483/1168) | | | | | | | | | | | |
| Fisher's exact test for biased strand distribution *p*-value = 7.83e-01 | | | | | | | | | | | |
| Kolmogorov-Smirnov test that lower quality scores support variant *p*-value = 7.53e-01 | | | | | | | | | | | |

Only 100 of 2655 total aligned reads displayed.  

`AAGTTCAGAATCAGATGAACGCATGGACGAAAGCTCAGGCCAAGGCTCTGGACGATTCCATGAAGTCAATGAACAAACTTGACGTAATCGACAAGCAATTCCAGAAGCGAATCAACGGTGAGTGGGTCTCAACGGATTTTAAGGATATGCCAGTCAACGAGAACACTGGTGAGTTCAAGCATAGCGATATGGTTAACTACGCCAATAAGAAGCTCGCTGAGATTGACAGTATGGACATTCCAGACGGTGCCAAGGATGCTATGAAGTTGAAGTACCTTCAAGCGGACTCTAAGGACGGAGCATTCCGTACAGCCATCGGAACCATGGTCACTGACGCTGGTCAAGAGTGGTCTGCCGCTGTGATTAACGGTAAGTTACCAGAACGAACCCCAGCTATGGATGCTCTGCGCAGAATCCGCAATGCTGACCCTCAGTTGATTGCTGCGCTATACCCAGACCAAGCTGAGCTATTCCTGACGATGGACATGATGGACAAGCAGGGTATTGACCCTCAGGTTATTCTTGATGCCGACCGACTGACTGTTAAGCGGTCCAAAGAGCAACGCTTTGAGGATGATAAAGCATTCGAGTCTGCACTGAAT  >  NC_001604.1/29397‑29998`  

                                                                                                                                                                                                                                                                                                            `|`                                                                                                                                                                                                                                                                                                               
  `gTTCAGAATCAGATGAACGCATGGACGAAAGCTCAGGCCAAGGCTCTGGACGATTCCATGAAGTCAATGAACAAACTTGACGTAATCGACAAGCAATTCCAGAAGCGAATCAACGGTGAGTGGGTCTCAACGGATTTTAAGGATATGCCAGTCAACGAGAACACTGGTGAGTTCAAGCATAGCGATATGGTTAACTACGCCAATAAGAAGCTCGCTGAGATTGACAGTATGGACATTCCAGACGGTGCCAAGGATGCTATGAAGTTGAAGTACCTTCAAGCGGACTCTAAGGACGGAGTAt                                                                                                                                                                                                                                                                                                             >  1:183461/1‑301 (MQ=255)`  
      `aGAATCAGATGAACGCATGGACGAAAGCTCAGGCCAAGGCTCTGGACGATTCCATGAAGTCAATGAACAAACTTGACGTAATCGACAAGCAATTCCAGAAGCGAATCAACGGTGAGTGGGTCTCAACGGATTTTAAGGATATGCCAGTCAACGAGAACACTGGTGAGTTCAAGCATAGCGATATGGTTAACTACGCCAATAAGAAGCTCGCTGAGATTGACAGTATGGACATTCCAGACGGTGCCAAGGATGCTATGAAGTTGAAGTACCTTCAAGCGGACTCTAAGGACGGAGTATTCCg                                                                                                                                                                                                                                                                                                         <  1:364300/301‑1 (MQ=255)`  
       `gAATCAGATGAACGCATGGACGAAAGCTCAGGCCAAGGCTCTGGACGATTCCATGAAGTCAATGAACAAACTTGACGTAATCGACAAGCAATTCCAGAAGCGAATCAACGGTGAGTGGGTCTCAACGGATTTTAAGGATATGCCAGTCAACGAGAACACTGGTGAGTTCACGCATAGCGATATGGTTAACTACGCCAATAAGAAGCTCGCTGAGATTGACAGTATGGACATTCCAGACGGTGCCAAGGATGCTATGAAGTTGAAGTACCTTCAAGAGGACTCTAAGGACGGAGAATTccct                                                                                                                                                                                                                                                                                                        >  1:69632/1‑299 (MQ=255)`  
       `gAATCAGATGAACGCATGGACGAAAGCTCAGGCCAAGGCTCTGGACGATTCCATGAAGTCAATGAACAAACTTGACGTAATCGACAAGCAATTCCAGAAGCGAATCAACGGTGAGTGGGTCTCAACGGATTTTAAGGATATGCCAGTCAACGAGAACACTGGTGAGTTCAAGCATAGCGATATGGTTAACTACGCCAATAAGAAGCTCGCTGAGATTGACAGTATGGACATTCCAGACGGTGCCAAGGATGCTATGAAGTTGAAGTACCTTCAAGCGGACTCTAAGGACGGAGTATTCCGt                                                                                                                                                                                                                                                                                                        >  1:495009/1‑301 (MQ=255)`  
           `cAGATGAAAGCATGGACGAAAGCTCAGGCCAAGGCTCTGGACGAGTCCATGAAGTCAATGAACAAACTTGACGTAATCGACAAGCAATTCCAGAAGCGAATCAACGGTGAGTGGGTCTCAACGGATTTTAAGGATATGCCAGTCAACGAGAACACTGGTGAGTTCAAGCATAGCGATATGGTTAACTACGCCAATAAGAAGCTCGCTGAGATTGACAGTATGGACATTCCAGACGGTGCCAAGGATGCTATGAAGTTGAAGTACCTTCAAGCGGACTCTAAGGACGGAGAATTCCGTACAg                                                                                                                                                                                                                                                                                                    <  1:335676/301‑1 (MQ=255)`  
             `gATGAACGCATGGACGAAAGCTCAGGCCAAGGCTCTGGATGATTCCATGAAGTCAATTAACAAACTTGACGTAATCGACAAGCAATTCCAGAAGCGAATCAACGGTGAGTGGGTCTCAACGGATTTTAAGGATATGCCAGTCAACGAGAACACTGGTGAGTTCAAGCATAGCGATATGGTTAACTACGCCAATAAGAAGCTCGCTGAGATTGACAGTATGGACATTCCAGACGGTGCCAAGGATGCTATGAAGTTGAAGTACCTTCAAGCGGACTCTAAGGACGGAGTATTCCgctgtctc                                                                                                                                                                                                                                                                                                  >  1:444525/1‑294 (MQ=255)`  
             `gATGAACGCATGGACGAAAGCTCAGGCCAAGGCTCTGGACGATTCCATGAAGTCAATGAACAAACTTGACGTAATCGACAAGCAATTCCAGAAGCGAATCAACGGTGAGTGGGTCTAAACGGATTTTAAGGATATGCCAGTCAACGAGAACACTGGTGAGTTCAAGCATAGCGCTATGGTTAACTACGCCAATAAGAAGCTCGCTGAGATTGACAGTATGGACATTCCAGACGGTGCCAAGGATTCTATGAAGTTGAAGTACCTTCAAGCGGACTCTAAGGACGGAGTATTCCGTACAGcc                                                                                                                                                                                                                                                                                                  >  1:213125/1‑301 (MQ=255)`  
             `gATGAAAGTAGGGACGAAATGTGAGGCAAAGGCTCTGGACGATTCCATGAAGTCAATGAAAAAACTGGACGTAATCGACAAGCAATTCCAGAAGCGAATCAACGGTGGGTGGGTCTCAACGGATTTTAAGGATATGCCAGTCAACGAGAACACTGGTGAGTTCAAGCATAGCGATATGGTTAACTACGCCAATAAGAAGCTCGCTGAGATTGACAGTATGTACATTCCAGACGGTGCCAAGGATGCTATGAAGTTGAAGTACCTTCAAGCGGACTCTAAGGACGGAGTATTCCGTACAGcc                                                                                                                                                                                                                                                                                                  <  1:298716/301‑1 (MQ=255)`  
                 `agagacaggAAGAAAGCTCAGGCCAAGGCTCTGGACGATTCCATGAAGTCAATGAACAAACTTGACGTAATCGACAAGCAATTCCAGAAGCGAATCAACGGTGAGTGTGTCTCAACGGATTTTAAGGATATGCCAGTCAACGAGAACACTGGTGAGTTCAAGCATAGCGATATGGTTAACTACTCCAATAAGAATCTCGCTGAGATTGACAGTATGGACATTCCAGACTGTGCCAAGGATGCTATGAAGTTGAAGTACCTTCAAGCGGACTCTAAGGACGGAGAATTCCGTACAGCCATCg                                                                                                                                                                                                                                                                                              <  1:240886/294‑1 (MQ=255)`  
                    `ggaTGGACGAAAGCTAGGTACAAGGCTCTGGACGATTCCAGGAAGAAAATGAAGAAAATTGACGTAATCGACAAGCAATTCCAGAAGAGAATCAACGGTGAGTGGGTCTCAACGGATTTTAAGGATATGCCAGTCAACGAGAACACTGGTGAGTTCAAGCATAGCGATATGGTTAACTACGCCAATAAGAAGCTCGCTGAGATTGACAGTATGGACATTCCAGACGGTGCCAAGGATGCTATGAAGTTGAAGTACCTTCAAGCGGACTCTAAGGACGGAGAATTCCGTACAGCCATCGGaa                                                                                                                                                                                                                                                                                           <  1:216501/299‑1 (MQ=255)`  
                          `aCGAAAGGTAAGGCGAAGGATCGGGAAGATTCCATGAAGGCAAAGAACAAACTTGACGTAATCGAGAAGAAATTCCAGAAGCGAATCAAAGGTGAGTGGGTCGCAACGGATTTTAAGGATATGCCAGTCAACGAGAACACTGGGGAGTTGAAGCATAGCGATATGGTTAACTACGCCAATAAGAAGCGAGCTGAGATTGACAGTATGGACATTCCAGACGGTGCCAAGGATGCTATGAAGTTGAAGTACCTTCAAGCGGACTCTAGGGACGGAGAATTACGTACAGCCATCGGAACCATgg                                                                                                                                                                                                                                                                                     <  1:195349/301‑1 (MQ=255)`  
                          `aCGAAAGCTCAGGCAAAGGCTCTGGACGATTCCATGAAGTCAATGAACAAACTTGACGTAATCGACAAGCAATTCCAGAAGCGAATCAACGGTGAGTGGGTCTCAACGGATTTTAAGGATATGCCAGTCAACGAGAACACTGGTGAGTTCAAGCATAGCGATATGGTTAACTACGCCAATAAGAAGCTCGCTGAGATTGACAGTATGGACATTCCAGACGGTGCCAAGGATGCTATGAAGTTGAAGTACCTTCAAGCGGACTCTAAGGACGGAGTATTCCGTACAGCCATCGGAACCATgg                                                                                                                                                                                                                                                                                     <  1:276290/301‑1 (MQ=255)`  
                           `cGAAAGCTCAGGCCAAGGCTCTGGACGATTCCATGAAGTCAATGAACAAACTTGACGTAATCGACAAGCAATTCCAGAAGCGAATCAACGGTGAGTGGGTCTCAACGGATTTTAAGGATATGCCAGTCAACGAGAACACTGGTGAGTTCAAGCATAGCGATATGGTTAACTACGCCAATAAGAAGCTCGCTGAGATTGACAGTATGGACATTCCAGACGGTGCCAAGGATGCTATGAAGTTGAAGTACCTTCAAGCGGACACTAAGGACGGAGAATTCCGTACAGCCATCGGAACCATGGt                                                                                                                                                                                                                                                                                    >  1:479362/1‑301 (MQ=255)`  
                           `cGAAAGCTCAGGCCAAGGCTCTGGACGATTCCATGAAGTCAATGAACAAACTTGACGTAATCGACAAGCAATTCCAGAAGCGAATCAACGGTGAGTGGGTCTCAACGGATTTTAAGGATATGCCAGTCAACGAGAACACTGGTGAGTTCAAGCATAGCGATATGGTTAACTACGCCAATAAGAAGCTCGCTGAGATTGACAGTATGGACATTCAAGACGGTGCCAAGGATGCTATGAAGTTGAAGTACCTTCAAGCGGACTCTAAGGACGGAGAATTCCGTACAGCCATCGGAACCATGGt                                                                                                                                                                                                                                                                                    >  1:414742/1‑301 (MQ=255)`  
                             `aaaGCTCAGGCCAAGGCTCTGTACGATTCCATGAAGTCAATTAACAAACTGGACGTAATCGACAAGCAATTCCAGAATCGAATCAACGATGAGTGGGTCTCAACGGATTTTAATGATATGCCAGTCAACGATAACACTGGTGAGTGCAAGCATATCGATATGGTTAACTACGCTAATAAGCAGCTCTCTGAGATGGACAGTATGGACATTCCAGACTGTGCCAAGGATGCTATGAAGTTGAAGTACCTTCAAGCGGACTCTAAGGACGGAGTATTCCGTACAGCCATCGGAACCATGGTCa                                                                                                                                                                                                                                                                                  <  1:64393/301‑1 (MQ=255)`  
                                    `aGGCCAAGGCGCCGGACGATTACATGAAGTCAATGAACAAACATGACGTAATCGACAAGCAATTCCAGAAGCGAATCAACGGTGAGTGGGTCTCAACGGATTTTAAGGATATGCCAGTCAACGAGAACACTGGTGAGTTCAAGCATAGCGATATGGTTAACTACGCCAATAAGAAGCTCGCTGAGATTGACAGTATGGACATTCCAGACGGTGCCAAGGATGCTATGAAGTTGAAGTACCTTCAAGCGGACTCTAAGGACGGAGAATTCCGTACAGCCATCGGAACCATGGTCACTGACGc                                                                                                                                                                                                                                                                           <  1:164662/301‑1 (MQ=255)`  
                                           `ggCTCTGGACGATTCCATGAAGTCAATGAACAAACTTGACGTAATCGACAAGCAATTCCAGAAGCGAATCAACGGTGAGTGGGTCTCAACGGATTTTAAGGATATGCCAGTCAACGAGAACACTGGTGAGTTCAAGCATAGCGATATGGTTAACTACGCCAATAAGAAGCTCGCTGAGATTTACAGTATGGACATTCCAGACGGTGCCAAGGATGCTATGAAGTTGAAGTACCTTAAAGCGGACTCTAAAGACGGAGTATTCCGTACAGCCATCGGAAACATGGTCACGTACGCTGGTCaa                                                                                                                                                                                                                                                                    >  1:290522/1‑301 (MQ=255)`  
                                            `gCTCTGGACGATTCCATGAAGTCAATGAACAAACTTGACGTAATCGACAAGCAATTCCAGAAGCGAATCAACGGTGAGTGGGTCTCAACGGATTTTAAGGATATGCCAGTCAACGAGAACACTGGTGAGTTCAAGCATAGCGATATGGTTAACTACGCCAATAAGAAGCTCGCTGAGATTGACAGTATGGACATTCCAGAAGGTGCCAAGGATGCTATGAAGTTGAAGTACCTTCAAGCGGACTCTAAGGACGGAGAATTCCGTACAGCCATCGGAACCATGGTCACTGACGCTGGTCAag                                                                                                                                                                                                                                                                   >  1:381730/1‑301 (MQ=255)`  
                                              `tctGGACGATTCCATGAAGTCAATGAACAAACTTGACGTAATCGACAAGCAATTCCAGAAGCGAATCAACGGTGAGTGGGTCTCAACGGATTTTAAGGATATGCCAGTCAACGAGAACACTGGTGAGTTCAATCATAGCGATATGGTTAACTACGCCAATAAGAAGCTCGCTGAGATTGACAGTATGGACATTCCAGACGGTGCCAAGGATGCTATGAAGTTGAAGTACCTTCAAGCGGACTCTAAGGACGGAGAATTCCGTACAGCCATCGGAACCATGGTCACTGACGCTGGTCAagag                                                                                                                                                                                                                                                                 <  1:226583/301‑1 (MQ=255)`  
                                               `ctGGACGATCCCATGAAGTCAATGAACAAACTTGACGTAATCGACAAGCAATTCCAGAAGCGAATCAACGGTGAGTGGGTATCAACGGATTTTAAGGATATGCCAGTCAACGAGAACACTGGTGAGTTCAAGCATAGCGATATGGTTAACTACGCCAATAAGAAGCTCGCTGAGATTGACAGTATGGACATTCCAGACGGTGCCAAGGATGCTATGAAGTTGAAGTACCTTCAAGCGGACTCTAAGGGCGGAGCATTCCGTACAGCCATCGGAACCATGGTCACTGACGCTGGTCAagagg                                                                                                                                                                                                                                                                >  1:424667/1‑300 (MQ=255)`  
                                                   `gcGATTCCATGAAGTCAATGAACAAACTTGACGTAATCGACAAGCAATTCCAGAAGCGAATCAACGGTGAGTGGGTCTCAACGGATTTTAAGGATCTGCCAGTCAACGAGAACACTGGTGAGTTCAAGCATAGCGATATGGTTAACTACGCCAATAAGAAGCTTGCTGAGATTGACAGTATGGACATTCCAGACGGTGCCAAGGATGCTATGAAGTTGAAGTACCTTCAAGCGGACTCTAAGGACGGAGTATTCCGTACAGCCATCGGAACCATGGTCACTGACGCTGGTCAAGAGTGGTc                                                                                                                                                                                                                                                            <  1:234682/300‑1 (MQ=255)`  
                                                      `aTTCCATGAAGTCAATGAACAAACTTGACGTAATCGACAAGCAATTCCAGAAGCGAATCAACGGTGAGTGGGTCTCAACGGATTTTAAGGATATGCCAGTCAACGAGAACACTGGTGAGTTCAAGCATAGCGATATGGTTAACTACGCCAATAAGAAGCTCGCTGAGATTGACAGTATGGACATTCCAGACGGTGCCAAGGATGCTATGAAGTTGAAGTACCTTCAAGCGGACTCTAAGGACGGAGTATTCCGTACAGCCATCGGAACCATGGTCACTGACGCTGGTCAAGAGTGGTCTGc                                                                                                                                                                                                                                                         >  1:100183/1‑301 (MQ=255)`  
                                                              `aaGTCAATGAACAAACTTGACGTAATCGACAAGCAATTCCAGAAGCGAATCAACGGTGAGTGGGTCTCAACGGATTTTAAGGATATGCCAGTCAACGAGAACACTGGTGAGTTCAAGCATAGCGATATGGTTAACTACGCCAATAAGAAGCTCGCTGAGATTGACAATATGGACATTCCAGACGGTGCCAAGGATGCTATGAAGTTGAAGTACCTTCAAGCGGACTCTAAGGACGGAGTATTCCGTACAGCCATCGGAACCATGGTCACTGACGCTGGTCAAGAGTGGTCTGCCGCtgtgt                                                                                                                                                                                                                                                 >  1:190240/1‑300 (MQ=255)`  
                                                                `gTCAATGAACAAACTTGACGTAATCGACAAGCAATTCCAGAAGCGAATCAACGGTGAGTGGGTCTCAACGGATTTTAAGGATATGCCAGTCAACGAGAACACTGGTGAGTTCAAGCATAGCGATATGGTTAACTACGCCAATAAGAAGCTCGCTGAGATTGACAGTATGGACATTCCAGACGGTGCCAAGGATGCTATTAAGTTGAAGTACCTTCAAGCGGACTCTAAGGACGGAGAATTCCGTACAGCCATCGGAACCATGGTCACTGACGCTGGTCAAGAGTGGTCTGCCGCTGTGAtt                                                                                                                                                                                                                                               >  1:169015/1‑301 (MQ=255)`  
                                                                   `aaTGAACAAACTTGACGTAACCGACAAGCAATTCCAGAAGCGAATCAACGGTGAGTGGGTCTCAACGGATTTTAAGGATAGGCCAGTCAACGAGAACACTGGTGAGTTCAAGCATAGCGATATGGTTAACTACGCCAATAAGAAGCTCGCTGAGATCGACAGTATGGACATTCCAGACGGTGCCAAGGATGCTATGAAGTTGAAGTACCTTCAAGCGGACTCTAAGGACGGAGAATTCCGTACAGCCATCGGAACCATGGTCACTGACGCTGGTCAAGAGTGGTCTGCCGCTGTGATTAAc                                                                                                                                                                                                                                            <  1:450048/301‑1 (MQ=255)`  
                                                                   `aaTGAACAAACTTGACGGAATCGACAACCAATTCCAGAAGCGAAGCAACGGTGAGTGGGTCTCAACGGATTTTAAGGAGATGCCATTCAACGAGAACACTGGTGAGTTCAAGCATAGCGATATGGTTAACTACGCCAATAAGAAGCTCGCTGAGATTGACAGTATGGACATTCCAGACGGTGCCAAGGATGCTATGAAGTTGAAGTACCTTCAAGCGGACTCTAAGGACGGAGAATTCAGTACAGAAATCGGAAAAAGGGGAAAGGAAGAGGGTAAAGAGTGGTCTGCCGCTGTGATTAAc                                                                                                                                                                                                                                            <  1:501856/301‑1 (MQ=255)`  
                                                                      `gacagaaCTTGAGGTAAACGACAAGCAATTCCAGAAGCGAATCAACGGTGAGTGGGTCTCAACGGATTTTAAGGATATGCCAGTCAACGAGAACACTGGTGAGTTCAAGCATAGCGATATGGTTAACTACGCCAATAAGAAGCTCGCTGAGATTGACAGTATGGACATTCCAGACGGTGCCAAGGATGCTATGAAGTTGAAGTACCTTCAAGCGGAATCTAAGGACGGAGAAGTCCGTACAGCCATCGGAACCATGGTCACTGACGCTGGTCAAGAGTGGTCTGCCGCTGTGATTAACGGt                                                                                                                                                                                                                                         <  1:17351/296‑1 (MQ=255)`  
                                                                            `acttgcattAATCAAAAAGCAAGGCAAGAAGCGAATAAACGGTGAGTGGGTCTCAACGGATTTTAAGGATATGCCAGTCAACGAGAACACTGGTGAGTTCAAGCATAGCGATATGGTTAACTACGCCAATAAGAAGCTCGCTGAGATTGACAGTATGGACATTCCAGACGGTGCCAAGGATGCTATGAAGTTGAAGTACCTTCAAGCGGACTCTAAGGACGGAGCATTCCGTACAGCCATCGGAACCATGGTCACTGACGCTGGTCAAGAGTGGTCTGCCGCTGTGATTAACGGTAAGTTa                                                                                                                                                                                                                                   <  1:281861/293‑1 (MQ=255)`  
                                                                                  `cGTAATCGACAAGCAAATCCAGAAGCGAATCAACGGTGAGTGGGTCTCAACGGATTTTAAGGATATGCCAGTCAACGAGAACACTGGTGAGTTCAAGCATAGCGATATGGTTAACTACGCCAATAAGAAGCTCGCTGAGATTGACAGTATGGACATTCCAGACGGTGCCAAGGATGCTATGAAGTTGAAGTACCTTCAAGCGGACTCTAAGGACGGAGAATTCCGTACAGCCATCGGAACCATGGTCACTGACGCTGGTCAAGAGTGGTCTGCCGCTGTGATTAACGGTAAGTTACCAgaa                                                                                                                                                                                                                             <  1:147260/301‑1 (MQ=255)`  
                                                                                              `gCAATTCCAGAAGCGAATCAACGGTGAGTGGGTCTCAACGGATTTTAAGGATATGCCAGTCAACGAGAACACTGGTGAGTTCAAGCATAGCGATATGGTTAACTACGCCAATAAGAAGCTCGCTGAGATTGACAGTATGGACATTCCAGACGGTGCCAAGGATGCTATGAAGTTGAAGTACCTTCAAGCGGACTCTAAGGACGGAGAATTCCGTACAGCCATCGGAACCATGGTCACTGACGCTGGTCAGGAGTGGTCTGCCGCTGTGATTAACGGTAAGTTACCAGAACGAACCCCAGCt                                                                                                                                                                                                                 >  1:150426/1‑301 (MQ=255)`  
                                                                                                  `ttCCAGAAGCGAATCAACGGTGAGTGGGTCTCAACGGATTTTAAGGATATGCCAGTCAACGAGAACACTGGTGAGTTCAAGCATAGCGATATGGTTAACTACGCCAATAAGAAGCTCGCTGAGATTGACAGTATGGACATTCCAGACGGTGCCAAGGATGCTATGAAGTTGAAGTACCTTCAAGCGGACTCTAAGGACGGAGTATTCCGTACAGCCATCGGAACCATGGTCACTGACGCTGGTCAAGAGTGGTCTGCCGCTGTGATTAACGGTAAGTTACCAGAACGAACCCCAGCTATgg                                                                                                                                                                                                             >  1:344813/1‑301 (MQ=255)`  
                                                                                                  `ttCCAGAAGCGAATCAACGGTGAGTGGGTCTCAACGGATTTTAAGGATATGCCAGTCAACGAGAACACTGGTGAGTTCAAGCATAGCGATATGGTTAACTACGCCAATAAGAAGCTCGATGAGATTGACAGTATGGACATTCCAGACGGTGCCAAGGATGCTATGAAGTTGAAGTACCTTCAAGCGGACTCTAAGGACGGAGTATTCCGTACAGCCATCGGAACCATGGTCACTGACGCTGGTCAAGAGTGGTCTGCCGCTGTGATTAACGGTAAGTTACCAGAACGAACCCCAGCTATgg                                                                                                                                                                                                             >  1:136844/1‑301 (MQ=255)`  
                                                                                                           `cGAATCAACGGTGAGTGGGTCTCAACGGATTTTAAGGATATGCCAGTCAACGAGAACACTGGTGAGTTCAAGCATAGCGATATGGTTAACTACGCCAATAAGAAGCTCGCTGAGATTGACAGTATGGACATTCCAGACGGTGCCAAGGATGCTATGAAGTTGAAGTACCTTCCAGCGGACTCTAAGGACGGAGAATTCCGTACAGCCATCGGAACCATGGTCACTGACGCTGGTCAAGAGTGGTCTGCCGCTGTGATTAACGGTAAGTTACCAGAACGAACCCCAGCTATGGATGCTCTgc                                                                                                                                                                                                    >  1:156376/1‑301 (MQ=255)`  
                                                                                                                  `aCGGTGAGTGGGTCTCAACGGATTTTAAGGATATGCCAGTCAACGAGAACACTGGTGAGTTCAAGCATAGCGATATGGTTAACTACGCCAATAAGAAGCTCGCTTAGATTGACAGTATGGACATTCCAGACGGTGCCAAGGATGCTATGAAGTTGAAGTACCTTCAAGCGGACTCTAAGGACGGAGTATTCCGTACAGCCATCGGAACCATGGTCACTGACGCTGGTCAAGAGTGGTCGGCCGCCGTGATTAACGGTAAGTTACCAGAACGACACCAAGATAAGGATGCTCAgcgacgaaa                                                                                                                                                                                             >  1:491183/1‑295 (MQ=255)`  
                                                                                                                  `aCGGTGAGTGGGTCTCAACGGATTTTAAGGATATGCCAGTCAACGAGAACACTGGTGAGTTCAAGCATAGCGATATGGTTAACTACGCCAATAAGAAGCTCGCTGAGATTGACAGTATGGACATTCCAGACGGTGCCAAGGATGCTATGAAGTTGAAGTACCTTCAAGCGGACTCTAAGGACGGAGTATTCCGTACAGCCATCGGAACCATGGTCACTGACGCTGGTCAAGAGTGGTCTGCCGCTGTGATTAACGGTAAGTTACCAGAACGAACCCCAGCTATGGAtctgtctcttataca                                                                                                                                                                                             >  1:405276/1‑287 (MQ=255)`  
                                                                                                                     `gTGAGTGGGTCTCAACGGATTTTAAGGATATGCCAGTCAACGAGAACACTGGTGAGTTCAAGCATAGCGATATGGTTAACTACGCCAATAAGAAGCTCGCTGAGATTGACAGTATGGACATTCCAGACGGTGCCAAGGATGCTATGAAGTTGAAGTACCTTCAAGCGGACTCTAATGACGGAGTATTCCGTACAGCCATCGGAACCATGGTCACTGACGCTGGTCAAGAGTGGTCTGCCGCTGTGATTAACGGTAAGTTACCAGAACGAACCCCAGCTATGGATGCTCTGCGCAGAATACg                                                                                                                                                                                          >  1:132673/1‑301 (MQ=255)`  
                                                                                                                     `gTGAGTGGGTCTCAACGGATTTTAAGGATATGCCAGTCAACGAGAACACTGGTGAGTTCAAGCATAGCGATATGGTTAACTACGCCAATAAGAAGCTCGCTGAGATTGACAGTATGGACATTCCAGACGGTGCCAAGGATGCTATGAAGTTGAAGTACCTTCAAGCGGACTCTAAGGACGGAGTATTCCGTACAGCCATCGGAACCATGGTCACTGACGCTGGTCAAGAGTGGTCTGCCGCTGTGATTAACGGTAAGTTAACAGAACGAACCCCAGCTATGGATGATCTGCGCAGAATCCg                                                                                                                                                                                          >  1:178782/1‑301 (MQ=255)`  
                                                                                                                      `tGAATGGGTCTAAACGGAATTTAAGGATGTGGCAGTCAAAGAGAACACTGGTGAGTTCAAGCAAAGCGATATGGTTAACTACGACAATAAGAAGCGCGCTGAGATTGACAGTATGGACATTACAGAAGGTGCCAAGGATGATATGAAGTTGAAGTACCTTCAAGAGGACTCTAAGGACGGGGTATTCCGTACAGCCATCGGAACCATGGTCACTGACGCTGGTCAAGAGTGGTCTGCCGCTGTGATTAACGGTAAGTTACCAGAACGAACCCCAGCTATGGATGCTCTGCGCAGAATCCGc                                                                                                                                                                                         <  1:105048/301‑1 (MQ=255)`  
                                                                                                                       `gaggtggTCTACACGGATTTTAAGGAAATGTCAGTCAACGAGAACACTGGTGAGTGCAAGCAGAGCGATATGGGTAACGACGCGAATAAGAAGCTCGCTGAGAGTGACAGTATGGACATTCCAGACGGTGACAAGGATGCTATTAAGTTGAAGTACCTTCAAGCGGACTCTAAGGACGGAGTATTCCGTACAGCCATCGGAACCATGGTTACTGACGCTGGTCAAGAGTGGTCTGCCGCTGTGATTAACGGTAAGTTACCAGAACGAACCCCAGCTATGGATGCTCTGCGCAGAATCCGCa                                                                                                                                                                                        <  1:484826/296‑1 (MQ=255)`  
                                                                                                                          `tGGGTCTCAACGGATTTTAAGGATATGCCAGTTAACGAGAACACTGGTGAGTTCAAGCATAGCGATATGGTTAACTACGCCAATAAGAAGCTCGCTGAGATTGACAGTATGGACATTCAAGACGGTGCCAAGGATGCTATGAAGTTGAAGTACCTTCAAGCGGACTCTAAGGACGGAGAATTCCGTACAGCCATCGGAACCATGGTCACTGACGCTGGTCAAGAGTGGTCTGCCGCTGTGATTAACGGTAAGTTACCAGAACGAA‑CCCAGCTATGGATGCTCTGCGCAGAATCCGCAATGc                                                                                                                                                                                    <  1:35363/301‑1 (MQ=255)`  
                                                                                                                                        `ttttAAGGATATGCCAGTCAACGAGAACACTGGTGAGTTCAAGCATAGCGATATGGTTAACTACGCCAATAAGCAGCTCGCTGAGATTGACAGTATGGACATTCCAGACGGTGCCAAGGATGCTATGAAGTTGAAGTACCTTCAAGCGGACTCTAAGGACGGAGTATTCCGTACAGCCATCGGCACCATGGTCACTGACGCTGGTCAAGAGTGGTCTGCCGCTGTGATTAACGGTAAGTTACCAGAACGAACCCCAGCTATGGATGCTCTTCGCAGAATCCGCAATGCTTACCCTAAGTTg                                                                                                                                                                       >  1:505038/1‑301 (MQ=255)`  
                                                                                                                                             `gacgactGCCAGTCAACGAGAACACTGGTGAGTTCAAGCATAGCGATATGGTTAACTACTCCAATAAGAAGCTCGCTGAGATTGACAGTATGGACATTCCAGACGGTGCCAAGGATGCTATGAAGTTGAAGTACCTTCAAGCGGACGCTAAGGACGGAGTATTCCGTACAGCCATCGGAACCATGGGCACTGACGCTGGTCAAGAGTGGTCTGCCGCTGTGATTAACGGTAAGTTACCAGAACGAACCCCAGCTATGGATGCTCTGCGCAGAATCCGCAATGCTGACCCTCAGTTGATtgc                                                                                                                                                                  <  1:95124/295‑1 (MQ=255)`  
                                                                                                                                                `atatGCCAGTCAACGAGAACACTGGTGAGTTCAAGCATAGCGATATGGTTAACTACGCCAATAAGAAGCTCGCTGAGATTGACAGTATGGACATTCCAGACGGTGCCAAGGATGCTATGAAGTTGAAGTACCTTCAAGCGGACTCTAAGGGCGGAGCATTCCGTACAGCCATCGGAACCATGGTCACTGACGCTGGTCAAGAGTGGTCTGCCGCTGTGATTAACGGTAAGTTACCAGAACGAACCCCAGCTATGGATGCTCTGCGCAGAATCCGAAATGCTGACCCTCAGTTGATtgctgc                                                                                                                                                               >  1:331259/1‑301 (MQ=255)`  
                                                                                                                                                       `agacAACGAGAACACTGGTGAGTTCAAGCATAGAGATATGGTTAACTACGCCAATAAGCAGCTCGCTGAGATTGACAGTATGGACATTCCAGACGGTGACAAGGATGCTATGAAGTTGAAGTACCTTCAAGCGGACTCTAAGGGCGGAGCATTCCGTACAGCCATCGGAACCATGGTCACTGACGCTGGTCAAGAGTGGTCTACCGCTGTGATTAACGGTAAGTAACCAGAACGAACCCCAGCTATGGATGCTCTGCGCAGAATCCGCAATGCTGACCCTCAGTTGATTGCTGCGCTATAc                                                                                                                                                        <  1:78664/298‑1 (MQ=255)`  
                                                                                                                                                         `tCAACGATAACACTGGTGAGTTCAAGCATAGCGATATGGTTAACTACGCCAATAATAAGCTCGCGGAGATTGACAGTATGGACATTCCAGACGGTGCCAAGGATGCTATGAAGTTGAAGTACCTTCAAGCGGACTCTAAGGACGGAGAATTCCGTACAGCCATCGGAACCATGGTCACTGACGCTGGTCAAGAGTGGTCTGCCGCTGTGATTAACGGTAAGTTACCAGAACGAACCCCAGCTATGGATGCTCTGCGCAGAATCCGCAATGCTGACCCTCAGTTGATTGCTGCGCTATAccc                                                                                                                                                      <  1:221708/301‑1 (MQ=255)`  
                                                                                                                                                         `acAACGAGAACACTGGTGAGTTCAAGCATATCGATATGGTTAACTACGCCAATAAGAAGCTCGCTGAGATTGACAGTATGGACATTCCAGACGGTGCCAAGGATGCTATGAAGTTGAAGTACCTTCAAGCAGAATCTAAGTAAGGAGTATTCCGTACAGCCATCGGAACCATGGTCACTGACGGTGGTCAAGAGTGGTCTTCCGCTGTGATTAACGGTAAGTTAGCAGATCGAACCCCAGCTATGGATGCTCTGCGCAGAATCCGCAATGCTGACCCTCAGTTGATTGCTGCGCTATAccc                                                                                                                                                      <  1:304163/300‑1 (MQ=255)`  
                                                                                                                                                             `agagaACACTGGTGAGTTCAAGCATAGCGATATGGTTAACTACGCCAATAAGAAGCTAGCTGAGATTGACAGTATGGACATTCCAGACGGTGCCAAGGATGCTATGAAGTTGAAGTACCTTCAATCGGACTCTAAGGGCGGAGCATTCCGTACAGCCATCGGAACCATGGTAACTGACGCTGGTCAAGAGTGGTCTGCCGCTGTGATTAACGGTAAGTTACCAGAACGAACCCCAGCTATGGATGCTCTGCGCAGAATCCGCAATGCTGACCCTCAGTTGATTGCTGCGCTATACCCAGAc                                                                                                                                                  <  1:315965/300‑1 (MQ=255)`  
                                                                                                                                                             `agagaACACTGGGGAGTTCAAGCATAGCGATATGGTTAACTACGCCAATAAGAAGCTCGCTGAGATTGACAGTATGGACATTCCAGACGGTGCCAAGGATGCTATGAAGTTGAAGTACCTTCAAGCGGACTCTAAGGACGGAGTATTCCGTACAGCCATCGGAACCATGGTCACTGACGCTGGTCAAGAGTGGTCTGCCGCTGTGATTAACGGTAAGTTACCAGAACGAACCCCAGCTATGGATGCTCTGCGCAGAATCCGCAATGCTGACCCTCAGTTGATTGCTGCGCTAGACCCAGAc                                                                                                                                                  <  1:87087/300‑1 (MQ=255)`  
                                                                                                                                                              `gagaACACTGGTGAGTTCAAGCATAGCGATATGGTTAACTACGCCAATAAGAAGCTCGCTGAGATTGACAGTATGGACATTCCAGACGGTGCCAAGGATGCTATGAAGTTGAAGTACCTTCAAGCGGACTCTAAGGACGGAGAATTCCGTACAGCCATCGGAACCATGGTCACTGACGCTGGTCAAGAGTGGTCTGCCGCTGTGATTAACGGTAAGTTACCAGAACGAACCCCAGCTATGGATGCTCTGCGCAGAATCCGCAATGCTGACCCTCAGTTGATTGCTGCGCTATACCCAGAcc                                                                                                                                                 <  1:464045/301‑1 (MQ=255)`  
                                                                                                                                                              `aagaACACTGGTGAGTTCAATCATAGCGATATGGTTAACTACGCCAATAAGAAGCTCGCTGAGATTGACAGTATGGACATTCCAGACGGTGCCAAGGATGCTATGAAGTTGAAGTACCTTCAAGCGGACTCTAAGGACTGAGAATTCCGTACAGCCATCGGAACCATGGTCACTGACGCTGGTCAAGAGTGGTCTGCCGCTGTGATTAACGGTAAGTTACCAGAACGAACCCCAGCTATGGATGCTCTGCGCAGAATCCGCAATGCTGACCCTCAGTTGATTGCTGCGCTATACCCAGAcc                                                                                                                                                 <  1:286911/300‑1 (MQ=255)`  
                                                                                                                                                                       `ggTGAGTTCAAGCATAGCGATATGGTTAACTACGCCAATAAGAAGCTCGCTGAGTTTGACAGTATGGACATTCCAGACGGTGCCAAGGATGCTATGAAGTTGAAGTACCTTCAAGCGGACTCTAAGGACGGAGTATTCCGTACAGCCATCGGAACCATGGTCACTGACGCTGGTCAAGAGTGGTCTGCAGCTGTGATTAACGGTAAGTTACCAGAACGAACCCCAGCTGTGGATGCTCTGCGCAGAATCCGCAATGCTGACCCTCAGTTGATTGCTGCGCTATACCCAGACCAAGCTGAGc                                                                                                                                        >  1:372772/1‑301 (MQ=255)`  
                                                                                                                                                                       `ggTGAGTTCAAGCATAGCGATATGGTTAACTACGCCAATAAGAAGCTCGCTGAGATTGACAGTATGGACATTCCAGACGGTGCCAAGGATGCTATGAAGTTGAAGTACCTTCAAGCGGACTCTAAGGACGGAGTATTCCGTACAGCCATCGGAACCATGGTCACTGACGCTGGTCAAGAGTGGTCTGCCGCTGTGATTAACGGTAAGTTACCAGAACGAACCCCAGCTATGGATGCTCTGCGCAGAATCCGCAATGCTGACCCTCAGTTGATTGCTGCGCTATACCCAGACCAAGCTGAGc                                                                                                                                        >  1:474104/1‑301 (MQ=255)`  
                                                                                                                                                                       `ggTGAGTTCAAGCATAGCGATATGGTTAACTACGCCAATAAGAAGCTCGCTGAGATTGACAGTATGGACATTCCAGACGGTGCCAAGGATGCTATGAAGTTGAAGTACCTTCAAGCGGACTCTAAGGACGGAGTATTCCGTACAGCCATCGGAACCATGGTCACTGACGCTGGTCAAGAGTGGTCTGCCGCTGTGATTAACGGTAAGTTACAAGAACGAACCCCAGCTATGGATGCTCTGCGCAGAATCCGCAATGCTGACCCTCAGTTGATTGCTGCGCTATACCCAGACCAAGCTGAGc                                                                                                                                        >  1:324683/1‑301 (MQ=255)`  
                                                                                                                                                                       `gacaggTTGAAGCAGAGCGATATGGTGAACTAAGCAAATAAGAATCTCGCTGAGATTGACAGTATGGACATCCCAGACGTTGGCAAGGATGCTATGAAGTTGAAGTACCTGCAAGCGGACTCTAAGGACGGAGAATTCCGTACAGCCATCGGAACGATGGTCACGGACGCTGGTCAAGAGTGGTCTGCCGCTGTGATTAACGGTAAGTTACCAGAACGAACCCCAGCTATGGATGCTCTGCGCAGAATCCGCAATGCTGACCCTCAGTTGATTGCTGCGCTATACCCAGACCAAGCTGAGc                                                                                                                                        <  1:16044/296‑1 (MQ=255)`  
                                                                                                                                                                            `tttCAAGCATAGCGATATGGTTAACTACGCCAATACGAAGCTCGCTGAGATTGACAGTATGTACATTCCAGACGGTTCCAAGGATGCTATGAAGTTGAAGTACCTTCAAGCGGACTCTAAGGACGGAGTATTCTGTACAGCCATCGGAACCATGGTCACTGACGCTGGTCAAGAGTGGTCTGCCGCTGTGATTAACGGTAAGTTACCAGAACGAACCCCAGCTATTGATGCTCTGCGCAGAATCCGCAATGCTGACCCTCAGTTGATTGCTGCGCTATACCCAGACAAAGCTGAGCTATTc                                                                                                                                   <  1:308416/300‑1 (MQ=255)`  
                                                                                                                                                                                 `aGCATAGCGATATGGTTAACTACGCCAATAAGAAGCTCGCTGAGATTGACAGTATGGACATTCCAGACGGTGCCAAGGATACTATGAAGTTGAAGTACCTTCAAGCGGACTCTAAGGACGGAGCATTCCGTACAGCCATCGGAACCATGGTCACTGACGCTGGTCAAGAGTGGTCTGCCGCTGTGATTAACGGTAAGTTACCAGAACGAACCCCAGCTATGGATGCTCTGCGCAGAATCCGCAATGCTGACCCTCAGTTGATTGCTGCGCTATACCCAGACCAAGCTGAGCTATTCCTGAc                                                                                                                              <  1:395538/301‑1 (MQ=255)`  
                                                                                                                                                                                 `aGCATAGCGATATGGTTAACTACGCAAATAAGAAGATCGCTGAGATTGACAGTATGGACATTCCAGACGGTGCCAAGGATGCTATGAAGTTGAAGTACCTTCAAGCGGACTCTAAGGGCGGAGCATTCCGTACAGCCATCGGAACCATGGTCACTGACGCTGGTCAAGAGTGGTCTGCCGCTGTGATTAACGGTAAGTTACCAGAACGAACCCCAGCTATGGATGCTCTGCGCAGAATCCGCAATGCTGACCCTCAGTTGATTGCTGCGCTATACCCAGACCAAGCTGAGCTATTCCTGAc                                                                                                                              <  1:208402/301‑1 (MQ=255)`  
                                                                                                                                                                                     `tAGCGATATGGTTAACTACGCCAATAAGAAGCTCGCTGAGATTGACAGTATGGACATTCCAGACGGTGCCAAGTATGCTATGAAGTTGAAGTACCTTCAAGCTGACTCTAAGGACGGAGAATTCCGTACAGCCATCGGAACCATGGTCACTGACGCTGGTCAAGAGTGGTCTTCCGCTGTGATTAACTGTAAGTTACCAGAACGAACCCCAGCTATGGATGCTCTGCGCAGAATCCGCAATGCTGACCCTCAGTTGATTGCTTCGCTATACCCAGACCAAGCTGAGCTATTccatgtccct                                                                                                                          >  1:386377/1‑293 (MQ=255)`  
                                                                                                                                                                                      `aGCGATAAGGTTAACGACGCCAATAAGAAGCTCGCTGAGATTGACAGAATGGACATTCCAGATGCTGCCAAGGATGCTATGAAGTTGAAGTACCTTCAAGCGGACTCTAAGGACGGAGTATTCCGTACAGCCATCGGAACCATGGTCACTGACGCTGGTCAAGAGTGGTCTGCCGCTGTGATTAACGGTAAGTTACCAGAACGAACCCCAGCTATGGATGCTCTGCGCAGAATCCGCAATGCTGACCCTCAGTTGATTGCTGCGCTATACCCAGACCAAGCTGAGCTATTCCTGACGATgg                                                                                                                         <  1:229973/301‑1 (MQ=255)`  
                                                                                                                                                                                       `gCGATATGGTTAACTACGCCAATAAGAAGCTCGCTGAGATTGACAGTATGGACATTCCAGACGGTGCCAAGGATGCTATGAAGTTGAAGTACCTTCAAGCGGACTCTAAGGACGGAGAATTCCGTACAGCCATCGGAACCATGGTCACTGACGCTGGTCAAGAGTGGTCTGCCGTTGTGATTAACGGTAAGTTACCAGAACGAAACCCAGCTATGGATGCTCTGCGCAGAATCTGAAATGCTGATCCTCAGTTGATTGAGGCGCTATACCCAGACCAAGCTGAGCTATTTCTGACGATGGa                                                                                                                        >  1:349848/1‑301 (MQ=255)`  
                                                                                                                                                                                             `gggTGAAATACGCCAATAAGAAGCTCGCTGAGATTGACAGTATGGACATTCCAGACGGTGCAAAGGATGCTATGAAGTTGAAGTACCTTCAAGCGGACTCTAAGGACGGAGAATTCCGTACAGCCATCGGAACCATGGTCACTGACGCTGGTCAAGAGTGGTCTGCCGCTGTGTTTAACGGTAAGTTACCAGAACGAACCCCAGCTATGGATGCTCTGCGCAGAATCCGCAATGCTGACCCTCAGTTGATTGCTGCGCTATACCCAGACCAAGCTGAGCTATTCCTGACGATGGACatgat                                                                                                                  <  1:339791/300‑1 (MQ=255)`  
                                                                                                                                                                                                  `aaCTACTCCAATAAGAAGCTCGCTGAGATTGACAGTATGGACATTCCAGACGGTGCCAAGGATGCTATGAAGTTGACGTACCTTCAAGCGGTCTCTAAGGACGGAGTATCCTGTACAGCCATCGCAACCATGGTGCCTGACGCTGGTCACGAGTGGACTGCCGCGGTGATTAATGGTAAGTTACCAGCACGAACCCCCGCTATGGATGCTATGTGCACAATCCGCAATGCTGTCCCTTGGTTGATTTCTGCGCTATACCCAGACCACGATGAGCTATTATTGACGGTGGACATGATGGACa                                                                                                             >  1:368037/1‑301 (MQ=255)`  
                                                                                                                                                                                                      `aCGACAATAAGAAGCGCACTGGGATTGACAGTAATGACATTCCAGACGGTGCCAAGGATGCTATGAAGTTGAAGTACCTGCAAGCGGACTATAAGGGCGGAGCATTCCGTACAGCCATCGGAACCATGGTCACTGACGCTGGTCAAGAGTGGTCTGCCGCTGGTATTAACGGTAAGTTACCAGAACGAACCCCAGCTATGGAGGCTCTGCGCAGAAGCCGCAATGCTGACCCTCAGGTGATTGCTGCGCTATACCCAGTCCAAGCTGAGCTATTCCTGACGATGGACATGATGGACAAGCa                                                                                                         <  1:245373/301‑1 (MQ=255)`  
                                                                                                                                                                                                       `acagaaTAAGAAGCTCGCTGTGATTGACAGGATGGACATTCCAGACGGTGCCAAGGATGCTATGAAGTTGAAGTACCGTCAAGCGGACTCTAAGGACGGAGCATGCCGTACAGCCATCGGAACCATGGTCACTGACGCTGGTCAAGAGTGGTCTTCCGCTGTGATTAACGGTACGTTACCAGAACGAACCCCAGCTATGGATGCTCTGCGCAGAATCCGCAATGCTGACCCTCAGTTGATTGCTGCGCTATACCCAGACCAAGCTGAGCTATTCCTGACGATGGACATGATGGACAAGCAg                                                                                                        <  1:55974/297‑1 (MQ=255)`  
                                                                                                                                                                                                         `ccAATAAGAAGCTCGCTGAGATTGACAGTATGGACATTCCAGACGGTGCCAAGGATGCTATGAAGTTGAAGTACCTTCAAGCGGACTCTAAGGACGGAGTATTCCGTACAGCCATCGGAACCATGGTCACTGACGCTGGTCAAGAGTGGTCTGCCGCTGTGATTAACGGTAAGTTACCAGAACGAACCCCAGCTATGGATGCTCTGCGCAGAATCCGCAATGCTGACCCTCAGTTGATTGCTGCGCTATACCCAGACCAAGCTGAGCTATTCCTGACGATGGACATGATGGACAAGCAggg                                                                                                      >  1:72829/1‑301 (MQ=255)`  
                                                                                                                                                                                                            `aTAAGAAGCTCGCTGAGATTGACAGTATGGACATTCCAGACGGTGCCAAGGATGCTATGAAGTTGAAGTACCTTCAAGCGGACTCTAAGGACGGAGTATTCCGTACAGCCATCGGAACCATGGTCACTGACGCTGGTCAAGAGTGGTCTGCCGCTGTGATTAACGGTAAGTTACCAGAACGAACCCCAGCTATGGATGCTCTGCGCAGAATCCGCAATGCTGACCCTCAGTTGATTGCTGCGCTATACCCAGACCAAGCTGAGCTATTCCTGACGATGGACATGATGGACAAGCAGGGTAt                                                                                                   >  1:60601/1‑301 (MQ=255)`  
                                                                                                                                                                                                            `aTAAGAAGCTCGCTGAGATTGACAGTATGGACATTCCAGACGGTGCCAAGGATGCTATGAAGTTGAAGTACCTTCAAGCGGACTCTAAGGACGGAGTATTCCGTACAGCCATCGGAACCATGGTCACTGACGCTGGTCAAGAGTGGTCTGCCGCTGTGATTAACGGTAAGTTACCAGAACGAACCCCAGCTATGGATGCTCTGCGCAGAATCCGCAATGCTGACCCTCAGTTGATTGCTGCGCTATACCCAGACCAAGCTGAGCTATTCCTGACGATGGACATGATGGACAAGCAGGGTAt                                                                                                   <  1:390644/301‑1 (MQ=255)`  
                                                                                                                                                                                                                `gaagCTCGCTGAGATTGACAGTATGGACATTCCATACGGTGCCAAGGATGCTATGAAGTTGAAGTACCTTCAAGCGGACTCTAAGGACGGAGTATTCCGTACAGCCATCGGAACCATGGTCACTGACGCTGGTCAAGAGTGGTCTGCCGCTGTGATTAACGGTAAGTTACCAGAACGAACCCCAGCTATGGATGCTCTGCGCAGAATCCGCAATGCTGACCCTCAGTTGATTGCTGCGCTATACCCAGACCAAGCTGAGCTATTCCTGACGATGGACATGATGGACAAGCAGGGTATTGAc                                                                                               <  1:114057/301‑1 (MQ=255)`  
                                                                                                                                                                                                                 `aagCTCGCTGAGATTGACAGTATGGACATTCCAGACGGTGCCAAGGATGCTATGAAGTTGAAGTACCTTCAAGCGGACTCTAAGGACGGAGTATTCCGTACAGCCATCGGAACCATGGTCACTGACGCTGGTCAAGAGTGGTCTGCCGCTGTGATTAACGGTAAGTTACCAGAACGAACCCCAGCTATGGATGCTCTGCGCAGAATCCGCAATGCTGACCCTCAGTTGATTGCTGCGCTATACCCAGACCAAGCTGAGCTATTCCTGACGATGGACATGATGGACAAGCAGGGTATTGAcc                                                                                              <  1:312350/301‑1 (MQ=255)`  
                                                                                                                                                                                                                          `gagaTTGACAGTATGGACATTCCAGACGGTGCCAAGGATGCTATGAAGTTGAAGTACCTTCAAGCGGACTCTAAGGGCGGAGCATTCCGTACAGCCATCGGAACCATGGTCACTGACGCTGGTCAAGAGTGGTCTGCCGCTGTGATTAACGGTAAGTTACCAGAACGAACCCCAGCTATGGATGCTCTGCGCAGAATCCGCAATGCTGACCCTCAGTTGATTGCTGCGCTATACCCAGACCAAGCTGAGCTATTCATGACGATGGACATGATGGACAAGCAGGGGATTGACCCTCAGGTTa                                                                                     >  1:457856/1‑301 (MQ=255)`  
                                                                                                                                                                                                                            `gaTTGACAGTATGGACATTCCAGACGGTGCCAAGGATGCTATGAAGTTGAAGTACCTTCAAGCGGACTCTAAGGACGTAGCATTCCGTACAGCCATCGGAACCATGGTCACTGACGCTGGTCAAGAGTGGTCTGCCGCTGTGATTAACGGTAAGTTACCAGAACGAACCCCAGCTATGGATGCTCTGCGCAGAATCCGCAATGCTGACCCTCAGTTGATTGCTGCGCTATACCCAGACCAAGCTGAGCTCTTCCTGCCGATGGACATTATGGACAAGCAGGGTATTGCCCCTCCGGTTCtt                                                                                   >  1:419132/1‑301 (MQ=255)`  
                                                                                                                                                                                                                            `gaTTGACAGTATGGACATTCCAGACGGTGCCAAGGATGCTATGAAGTTGAAGTACCTTCAAGCGGACTCTAAGGACGGAGTATTCCGTACAGCCATCGGAACCATGGTCACTGACGCTGGTCAAGAGTGGTCTGCCGCTGTGATTAACGGTAAGTTACCAGAACGAACCCCAGCTATGGATGCTCTGCGCAGAATCCGCAATGCTGACCCTCAGTTGATTGCTGCGCTATACCCAGACCAAGCTGAGCTATTCCTGACGATGGACATGATGGTCAAGCAGGGTATTGACCCTCAGGTTAtt                                                                                   >  1:439713/1‑301 (MQ=255)`  
                                                                                                                                                                                                                            `gaTTGACAGTATGGACATTCCAGACGGTGCCAAGGATGCTATGAAGTTGAAGTACCTTCAAGCGGACTCTAAGGACGGAGTATTCCGTACAGCCATCGGAACCATGGTCACTGACGCTGGTCAAGAGTGGTCTGCCGCTGTGATTAACGGTAAGTTACCAGAACGAACCCCAGCTATGGAGGCTCTGCGCAGAATCCGCAATGCTGACCCTAAGTGGATTGATGCGATATACACAGACCAAGCTGAGCTATTACTGACGAGGGACATGATGGAAAAGCAGGGTATTGACACTCAGGTTCtt                                                                                   >  1:510010/1‑301 (MQ=255)`  
                                                                                                                                                                                                                            `gaTTGACAGTATGGACATTCCAGACGGTGCCAAGGATGCTATGAAGTTGAAGTACCTTCAAGCGGACTCTAAGGACGGAGTATTACGTACAGCCATCGGAACCATGGTCACTGACGCTGGTCAAGAGTGGTCTGCCGCTGTGATTAACGGTAAGTTACCAGAACGAACCCCAGCTATGGATGCTCTGCGCAGAATCCGCAATGCTGACCCTCAGTTGATTGCTGCGATATACCCAGACCAAGCTGAGCTATTCCTGACGATGGACATGATGGACAAGCAGGGTATTGACCCTCAGGTTAtt                                                                                   >  1:434484/1‑301 (MQ=255)`  
                                                                                                                                                                                                                              `ttGACAGTATGGACATTCCAGACGGTGCCAAGGATGCTAGGAAGTTGAAGTACCTTCAAGCGGACTCTAAGGACGGAGTATTCCGTACAGCCATCGGAACCATGGTCACTGACGCTGGTCAAGAGTGGTCTGCCGCTGTGATTAACGGTAAGTTACCATAACGAACCCCAGCTATGGATGCTCTGCGCAGAATCCGCAATGCTGACCCTCAGTTGATTGCTGCGCTATACCCAGACCAAGCTGAGCTATTCCTGACGATGGACATGATGGACAAGCAGGGTATTGACCCTCAGGTTATTCt                                                                                 <  1:262617/301‑1 (MQ=255)`  
                                                                                                                                                                                                                                   `aGTATGGACATTCCAGACGGTGCCAAGGATGCTATGAAGTTGAAGTACCTTCAAGCGGACTCTAAGGACGGAGAATTCCGTACAGCCATCGGAACCATGGTCACTGACGCTGGTCAAGCGTGGTCTGCCGCTGTGATTAACGGTAAGTTACCAGAACGAACCCCAGATATGGATGCTCTGCGCAGAATCCGCAATGCTGACCCTCAGTTGATTGCTGCGCTATACACAGACCAAGCTGAGCTATTCCTGACGATGGAAATGATGGACAAGCAGGGACTTGACCCtgtctcttatacacatc                                                                            >  1:320392/1‑285 (MQ=255)`  
                                                                                                                                                                                                                                    `gTATGGACATTCCAGACGGTGCCAAGGATGCTATGAAGTTGAAGTACCTTCAGGCGGACTCTAAGGACGGAGTATTCCGTACAGCCATCGGAACCATGGTCACTGACGCTGGTCAAGAGTGGTCTGCCGCTGTGATTAACGGTAAGTTACCAGAACGAACCCCAGCTATGGATGCTCTGCGCAGAATCCGCAATGCTGACCCTCAGTTGATTGCTGCGCTATACCCAGACCAAGCTGAGCTATTCCTGACGATGGACATGATGGACAAGCAGGGTATTGACCCTCAGGTTATTCTTGATGc                                                                           <  1:200301/301‑1 (MQ=255)`  
                                                                                                                                                                                                                                    `gTATGGACATTCCAGACGGTGCCAAGGATGCTATGAAGTTGAAGTACCTTCAAGCGGACTCTAAGGACGGAGTATTCCGTACAGCCATCGGAACCATGGTCACTGACGCTGGTCAAGAGTGGTCTGCCGCTGTGATTAACGGTAAGTTACCAGAACGAACCCCAGCTATGGATGCTCTTCGCAGAATCCGCAATGCTGACCCTCAGTGGATTGCTGCGCTATACCCAGACCAAGCTGAGCTATTCCTGACGATGGACATGATGGACAAGCAGGGTATTGACCCTCAGGTTATTCTTGATGc                                                                           >  1:271399/1‑301 (MQ=255)`  
                                                                                                                                                                                                                                    `gTATGGACATTCCAGACGGTGCCAAGGATGCTATGAAGTTGAAGTACCTTCAAGCGGACTCTAAGGACGGAGAATTCCGTACAGCCATCGGAACCATGGTCACTTACGCTGGTCAAGAGTGGTCTGCCGCTGTGATTAACGGTAAGTTACCAGAACGAACCCCAGCTATGGATGCTCTGCGCAGAATCCGCAATGCTGACCCTCAGTTGATTGCTGCGCTATACCCAGACCAAGCTGAGCTATTCCTGACGATGGACATGATGGACAAGCAGGGTATTGACCCTCAGGTTATTCTTGATGc                                                                           >  1:253914/1‑301 (MQ=255)`  
                                                                                                                                                                                                                                    `gTATGGACATTCCAGACGGTGCCAAGGATGCTATGAAGTTGAAGTACCTTCAAGCGGACTCTAAGGACGGAGAATTCCGTACAGCCATCGGAACCATGGTCACTGACGCTGGTCAAGAGTGGTCTGCCGCTGTGATTAACGGTAAGTTACCAGAACGAACCCCAGCTATGGATGCTCTGCGCAGAATCCGCAATGCTGACCCTCAGTTGATTGCTGCGCTATACCCAGACCAAGCTGAGCTATTCCTGACGATGGACATGATGGACAAGCAGGGTATTGACCCTCAGGTTATTCTTGATGc                                                                           <  1:26718/301‑1 (MQ=255)`  
                                                                                                                                                                                                                                      `aTGGACATTCCAGACGGTGCCAAGGATGCTATGAAGTTGAAGTACCTTCAAGCGGACTCTAAGGACGGAGAATTCCGTACAGCCATCGGAACCATGCTCACTGACGCTGGTCAAGATTGGTCTGCCGCTGTGATTAACGGTAAGTTACCAGAACGAACCCCAGCTATGGATGCTCTGCGCAGAATCCTCAATGCTGACCCTCAGTTGATTGCTGCGCTATACCAAGACCAAGCTGAGCTATTCCTGACGATGGACCTGATGGACAAGCAGGGTATTGACCCTCAGGTTATTCTTGATGccg                                                                         >  1:127424/1‑301 (MQ=255)`  
                                                                                                                                                                                                                                             `ttCCAGACGGTGCCAAGGATGCTATGAAGTTGAAGTACCTTCAAGCGGACTCTAAGGACGGAGAATTCCGTACAGCCATCGGAACCATGGTCACTGACGCTGGTCAAGAGTGGTCTGCCGCTGTGATTAACGGTAAGTTACCAGAACGAACCCCAGCTATGGATGCTCTGCGCAGAATCCGCAATGCTGACCCTCAGTTGATTGCTGCGCTATACCCAGACCAAGCTGAGCTATTCCTTACGATGGACATGATGGACAAGCAGGGTATTGACCCTCAGGTTATTCTTGATGCCGACCgact                                                                  >  1:487037/1‑301 (MQ=255)`  
                                                                                                                                                                                                                                              `tCCAGACGGTGCCAAGGATGCTATGAAGTTGAAGTACCTTCAAGCGGACTCTAAGGACGGAGTAGTCCGTACAGCCATCGGAACCATGGTCACTGACGCTGGTCAAGAGTGGTCTGCCGCTGTGATTAACGGTAAGTTACCAGAACGAACCCCAGCTATGGATGCTCTTCGCAGAATCCGCAATGCTGACCCTCAGTTGATTGCTGCGCTATACCCAGACCAAGCTGAGCTATTCCTGACGATGGACATGATGGACAAGCAGGGTATTGACCCTCAGGTTATTCTTGATGCCGACCgactg                                                                 <  1:468973/301‑1 (MQ=255)`  
                                                                                                                                                                                                                                                `cagtaggTGCCGAGGATGCTATGAAGTTGAAGTACCTTCAAGCGGACTCTAAGGACGGAGTATTCCGTACAGCCATCGGAACCATGGTCACTGACGCTGTTCAAGAGTGGTCTGCCGCTGTGATTAACGGTAAGTTACCAGAACGAACCCCAGCTATGGATGCTCTGCGCAGGATCCGCAATGCTGACCCTCAGTTGATTGCTGCGCTATACCCAGACCAAGCTGAGCTATTCCTGACGATGGACATGATGGACAAGCAGGGTATTGACCCTCAGGTTATTCTTGATGCCGACCgactgac                                                               <  1:257667/296‑1 (MQ=255)`  
                                                                                                                                                                                                                                                      `gTGCCAAGGATGCTATGAAGTTGAAGTACCTTCAAGCGGACTCTAAGGACGGAGAATTCCGTACAGCCATCGGAACCATGGTCACTGACGCTGGTCAAGAGTGGTCTGCCGCTGTGATTAACGGTAAGTTACCAGAACGAACCCCAGCTATGGATGCTCTGCGCAGAATCCGCAATGCTGACCCTCAGTTGATTGCTGCGCTCTACCCAGACCAAGCTGAGCTATTCCTGACGATGGACATGATGGACAAGCAGGGTATTGACCCTCAGGTTATTCTTGATGCCGACCGACTGACTGTTaa                                                         >  1:83824/1‑301 (MQ=255)`  
                                                                                                                                                                                                                                                       `gatgtgtataagagacaggttgaagtACCTTCAAGCGGACTCTAAGGACGGAGAATTCCGTACAGCCATCGGAACCATGGTCACTGACGCTGGTCAAGAGTGGTCTGCCGCTGTGATTAACGGTAAGTTACCAGAACGAACCCCAGCTATGGATGCTCTGCGCAGAATCCGCAATGCTGACCCTCAGTTGATTGCTGCGCTATACCCAGACCAAGCTGAGCTATTCCTGACGATGGACATGATGGACAAGCAGGGTATTGACCCTCAGGTTATTCTTGATGCCGACCGACTGACTGTTAAg                                                        <  1:122781/283‑1 (MQ=255)`  
                                                                                                                                                                                                                                                          `cAAGGATGCTATGAAGTTGAAGTACCTTCAAGCGGACTCTAAGGGCGGAGCATTCCGTACAGCCATCGGAACCATGGTCACTGACGCTGGTCAAGAGTGGTCTGCCGCTGTGATTAACGGTAAGTTACCAGAACGAACCCCAGCTATGGATGCTCTGCTCAGAATCCGCAATGCTGACCCTCAGTTGATTGCTGCGCTATACCCAGACCAAGCTGAGCTATTCCTGACGATGGACATGATGGACAAGCAGGGTATTGACCCTCAGGTTATTCTTGATGCCGACCGACTGACTGTTAAGCgg                                                     <  1:90676/301‑1 (MQ=255)`  
                                                                                                                                                                                                                                                          `cAAGGATCATATAATGTTGAAGTACCTTCAAGCGGACTCTAAGGACGGAGTATACCGTAACGCCATCGTAACCAGGGTCACTGACGCTGGTCAAGAGTGGTCTGCCACTGTGATTAACGGTAAGTTACCAGAACGAACCCCAGCTATGGATGCTCTGCGCAGAATCCGCAATGCTGACCCTCAGTTGATTGCTGCGCTATACCCAGACCAAGCTGAGCTATTCCTGACGATGGACATGATGGACAAGCAGGGTATTGACCCTCAGGTTATTCTTGATGCCGACCGACTGACTGTTAAGCgg                                                     <  1:497695/301‑1 (MQ=255)`  
                                                                                                                                                                                                                                                                `tGCTATGAAGTTGAAGTACCTTCAAGCGGACTCTAAGGACGGAGAATTCCGTACAGCCATCGGAACCATGGTCACTGACGCTGGTCAAGAGTGGTCTGCCGCTGTGATTAACGGTAAGTTACCAGAACGAACCCCAGCTATGGATGCTCTGCGCAGAATCCGCAATGCTGACCCTCAGTTGATTGCTGCGCTATACCCAGACCAAGCTGAGATATTCATGACGATGGACATGATGGACAAACAGGGTATTGACACTCAGGTTATTCTTGATGCCGACCGACTGCCTGTTACGCTGTCCcaa                                               >  1:295322/1‑301 (MQ=255)`  
                                                                                                                                                                                                                                                                  `cTATGAAGTTGAAGTACCTTCAAGCGGACTCTAAGGACGGAGTATTCCGTACAGCCATCGGAACCATGGTCACTGACGCTGGTCAAGAGTGGTCTGCCGCTGTGATTAACGGTAAGTTACCAGAACGAACCCCAGCTATGGATGCTCTGCGCAGAATCCGCAATGCTGACCCTCAGTTGATTGCTGCGCTATACCCAGACCAAGCTGAGCTATTCCTGACGATGGACATGATGGACAAGCAGGGTATTGACCCTCAGGTTATTCTTGATGCCGACCGACTGACTGTTAAGCGGTCCAAaga                                             >  1:204841/1‑301 (MQ=255)`  
                                                                                                                                                                                                                                                                  `cTATGAAGTTGAAGTACCTTCAAGCGGACTCTAAGGACGGAGTATTCCGTACAGCCATCGGAACCATGGTCACTGACGCTGGTCAAGAGTGGTCTGCCGCTGTGATTAACGGTAAGTTACCAGAACGAACCCCAGCTATGGATGCTCTGCGCAGAATCCGCAATGCTGACCCTCAGTTGATTGCTGCGCTATACCCAGACCAAGCTGAGCTATTCCTGACGATGGACATGATGGACAAGCAGGGTATTGACCCTCAGGTTATTCTTGATGCCGACCGACTGACTGTTAAGCGGTCCAAaga                                             <  1:142932/301‑1 (MQ=255)`  
                                                                                                                                                                                                                                                                               `gtACCTTCAAGCGGACTCTAAGGACGGAGAATTCCGTACAGCCATCGGAACCATGGTCACTGACGCTGGTCAAGAGTGGTCTGCCGCTGTGATTAACGGTAAGTTACAAGAACGAACCCCAGCTATGGATGCTCTGCGCAGAATCCGCAATGCTGACCCTCAGTTGATTGCTGCGCTATACCCAGACCAAGCTGAGCTATTCCTGACGATGGACATGATGTACAAGCAGGGAATTGACCCTCAGGTTATTCTTGATGCCGACCGACTGACTGTTAAGCGGTCCAAAGAGCAACGCTTTGAg                                >  1:410647/1‑301 (MQ=255)`  
                                                                                                                                                                                                                                                                                 `tcggagatgtgtataagagacagggAGTATTCCGTACAGCCATCGGAACCATGGTCACTGACGCTGGTCAAGAGTGGTCTGCCGCTGTGATTAATGGTAAGTTACCAGAACGAACCCCAGCTATGGATGCTCTGCGCAGAATCCGCAATGCTGACCCTCAGTTGATTGCTGCGCTATACCCAGACCAAGCTGAGCTATTCCTGACGATGGACATGATGGACAAGCATGGTATTGACCCTCAGGTTATTCTTGATGCCGACCGACTGACTGTTAAGCGGTCCAAAGAGCAACGCTTTGAGga                              <  1:453205/278‑1 (MQ=255)`  
                                                                                                                                                                                                                                                                                   `cTTCAAGCGGACTCTAAGGACGGAGTATTCCGTACAGCCATCGGAACCATGGTCACTGACGCTGGTCAAGAGTGGTCTGCCGCTGTGATTAACGGTTAGTTACCAGAACGAACCCCAGCTCTGGATGCTCTGCGCAGAATCCGCAATGCTGACCCTCAGTTGATTGCTGCGCTATCCCCAGCCCAAGCTGAGCTCTTCCTGACGTTGGTCCCGATGGCCAATCAGGGTATTGACCCTCAGGTTCTTCTTGATTCCGCCCGACTGCCTCTTAATCCGTCCCAATAGCAACGCTTTTAGgatg                            >  1:430652/1‑301 (MQ=255)`  
                                                                                                                                                                                                                                                                                     `gtataagagacagaaGGATGGAGAATTCCGTACAGCCATCGGAACCATGGTCACTGACGCTGGTCAAGAGTTGTCTGCCGCTGTGATTAACGGTAAGTTACCAGAACGAACCCCAGCTATGGATGCTCTGCGCAGAATCCGCAATGCTGACCCTCAGTTGATTGCTGCGCTATACCCAGACCAAGCTGAGCTATTCCTGACGATGGACATGATGGACAAGCAGGGTATTGACCCTCAGGTTATTCTTGATGCCGACCGACTGACTGTTAAGCGGTCCAAAGAGCAACGCTTTGAGgatgat                          <  1:118923/288‑1 (MQ=255)`  
                                                                                                                                                                                                                                                                                              `ctctAAGGACGGAGTATTCCGTACAGCCATCGGAACCATGGTCACTGACGCTGGTCAAGAGTGGTCTGCCGCTGTGATTAACGGTAAGTTACCAGAACGAACCCCAGCTATGGATGCTCTGCGCAGAATCCGCAATGCTGACCCTCAGTTGATTGCTGCGCTATACCCAGACCAAGCTGAGCTATTCCTGACGATGGACATGATGGACAAGCAGGGTATTGACCCTCAGGTTATTCTTGATGCCGACCGACTGACTGTTAAGCGGTCCAAAGAGCAACGCTTTGAGGATGATAAAGCATTc                 >  1:357882/1‑301 (MQ=255)`  
                                                                                                                                                                                                                                                                                                `ctACGGACGGAGCATTCCGTACAGCCATCGGAACCATGGTCACTGACGCTGGTCAAGAGTGGTCTGCCGCTGTGATTAACGGTAAGTTACCAGAACGAACCCCAGCTATGGATGCTCTGCGCAGAATACGCAATGCTGACCCTCAGTTGATTGCTGCGCTATACCCAGACCAAGCTGAGCTATTCCTGACGATGGACCTGATGGTCAAGCAGGGTATTGACCCTAAGGTTATTCTTGATGCCGACCGACTGACTGTTAAGCGGTCCAAAAAGCAAAGCTTTGAGGATGATAACGcctgtct               >  1:109828/1‑295 (MQ=255)`  
                                                                                                                                                                                                                                                                                                `ctAAGGACGGAGTATTCCGTACAGCCATCGGAACCATGGTCACTGACGCTGGTCAAGAGTGGTCTGCCGCTGTGATTAACGGTAAGTTACCAGAACGAACCCCAGCTATGGATGCTCTGCGCAGAATCAGCAATGCTGACACTCAGTTGATTGCTGCGCTATACCCAGACCAAGCTGAGCTATCCCTGACGATGGACATGATGGACAAGCAGGGTATTGACCCTCAGGTTATTCTTGATGCCGACCGACTGACTGTTAAGCGGTCCAAAGAGCAACGCTTTGAGGATGAAAAAGCATTCgg               >  1:377573/1‑300 (MQ=255)`  
                                                                                                                                                                                                                                                                                                    `ggACGGAGTATTCCGTACAGCCATCGGAACCATGGTCACTGACGCTGGTCAAGAGTGGTCTGCCGCTGTGATTAACGGTAAGTTACCAGAACGAACCCCAGCTATGGATGCTCTGCGCAGAATCCGCAATGCTGACCCTCAGTTGATTGCTGCGCTATACCCAGACCAAGCTGAGCTATTCCTGACGATGGACATGATGGACAAGCAGGGTATTGACCCTCAGGTTATTCTTGATGCCGACCGACTGACTGTTAAGCGGTCCAAAGAGCAACGCTTTGAGGATGATAAAGCATTagagcgg           >  1:399199/1‑298 (MQ=255)`  
                                                                                                                                                                                                                                                                                                        `ggAGAATTCCGTACAGCCATCGGAACCATGGTCACTGACGCTGGTCAAGAGTGGTCTGCCGCTGTGATTAACGGTAAGTTACCAGAACGAACCCCAGCTATGGATGCTCTGCGCAGAATCCGCAATGCTGACCCTCAGTTGATTGCTGCGCTATACCCAGACCAAGCTGAGCTATTCCTGACGATGGACATGATGGACAAGCAGGGTATTGACCCTCAGGTTATTCTTGATTCCGACCGACTGTCTGTTAAGCGGTCCAAAGAGCAACGCTTTGAGGATGATAAAGCATTCGAGTCTGCAc       >  1:250215/1‑301 (MQ=255)`  

                                                                                                                                                                                                                                                                                                            `|`                                                                                                                                                                                                                                                                                                               
`AAGTTCAGAATCAGATGAACGCATGGACGAAAGCTCAGGCCAAGGCTCTGGACGATTCCATGAAGTCAATGAACAAACTTGACGTAATCGACAAGCAATTCCAGAAGCGAATCAACGGTGAGTGGGTCTCAACGGATTTTAAGGATATGCCAGTCAACGAGAACACTGGTGAGTTCAAGCATAGCGATATGGTTAACTACGCCAATAAGAAGCTCGCTGAGATTGACAGTATGGACATTCCAGACGGTGCCAAGGATGCTATGAAGTTGAAGTACCTTCAAGCGGACTCTAAGGACGGAGCATTCCGTACAGCCATCGGAACCATGGTCACTGACGCTGGTCAAGAGTGGTCTGCCGCTGTGATTAACGGTAAGTTACCAGAACGAACCCCAGCTATGGATGCTCTGCGCAGAATCCGCAATGCTGACCCTCAGTTGATTGCTGCGCTATACCCAGACCAAGCTGAGCTATTCCTGACGATGGACATGATGGACAAGCAGGGTATTGACCCTCAGGTTATTCTTGATGCCGACCGACTGACTGTTAAGCGGTCCAAAGAGCAACGCTTTGAGGATGATAAAGCATTCGAGTCTGCACTGAAT  >  NC_001604.1/29397‑29998`

  

Alignment Legend

Aligned base mismatch/match (shaded by quality score): `ATCG/ATCG` `< 3 ≤` `ATCG/ATCG` `< 10 ≤` `ATCG/ATCG` `< 21 ≤` `ATCG/ATCG` `< 34 ≤` `ATCG/ATCG` `< 38 ≤` `ATCG/ATCG`

Unaligned base: `atcg`    Masked matching base: `atcg`    Alignment gap: `‑`    Deleted base: `‑`
